# Performance of game sessions in VR vs standard 2D monitor environment. an EEG study

**DOI:** 10.3389/fphys.2024.1457371

**Published:** 2024-10-22

**Authors:** Urszula Malinowska, Jakub Wojciechowski, Marek Waligóra, Jacek Rogala

**Affiliations:** ^1^ Institute of Experimental Physics, University of Warsaw, Warsaw, Poland; ^2^ Nencki Institute of Experimental Biology, Warsaw, Poland; ^3^ Bioimaging Research Center, Institute of Physiology and Pathology of Hearing, Kajetany, Poland; ^4^ Faculty of Physics, University of Warsaw, Warsaw, Poland

**Keywords:** virtual reality, EEG, DMTS, cognitive rehabilitation, time-frequency

## Abstract

Nowadays studies using Virtual Reality (VR) are gaining high popularity due to VR being a better approximation of the ecological environment for visual experiments than standard 2D display settings. VR technology has been already applied in medicine in the therapy of mental disorders, neurorehabilitation, and neurofeedback. However, its effectiveness compared to the standard 2D procedure is still not fully documented and limited information about the neurophysiological underpinnings of VR is provided. In this study, we tested participants’ performance during several sessions of the computer game in two different environments, VR vs. 2D monitor display. Participants performed three 25 min gaming sessions of adapted Delay Match-To-Sample task during EEG recording. The results showed that the VR group outperformed the 2D display group in the first session and then maintained its performance level throughout the remaining two sessions while the 2D group increased performance in each session eventually leveling up in the last one. Also group differences in the EEG activity were most profound only in the first session. In this session, the VR group was characterized by stronger and more synchronized neuronal activity, especially in delta, theta, and gamma bands. The VR group was less impacted by visual arousals as indicated by the theta/beta2 ratio in parietal electrodes.

## 1 Introduction

Improving the effectiveness of treatments for mental disorders has been and continues to be a key issue in medicine, especially with the significant increase in the prevalence of nervous system dysfunction, not only due to dementia, trauma, or neurodegenerative diseases but also disorders such as Attention deficit hyperactivity disorder (ADHD), which is increasingly prevalent in younger people (Oehrlein et al., 2016). The use of new technological advances may allow for better outcomes while reducing the duration or number of cumbersome therapy sessions. With the rapid development of Virtual Reality (VR) technology, there are more and more reports on research and clinical applications (Wright, 2014; Parsons et al., 2013). Researchers have demonstrated the effectiveness of VR in therapeutic applications, and VR systems have been used in the treatment of many psychiatric disorders, pain management, and neurorehabilitation for several years (Bohil et al., 2011). Studies on brain function in VR environments mostly related to passive watching of 3D videos indicate that the fully immersive 3D-enriched environment requires the allocation of more brain and sensory resources for cognitive/motor control than 2D presentations (Slobounov et al., 2015). In another EEG-VR study (Malik et al., 2015), Malik et al. reported higher EEG absolute power across various brain regions during 3D video watching, especially including the occipital region in the theta band and the frontal and parietal regions in the alpha band, while in fMRI, Chen et al. (2015) observed increased visual fatigue caused by watching 3D TV compared to 2D. Convergent results on increased load in VR settings are also shown by studies using interactive game or simulator environments. For example, Kakkos and colleagues’ findings (Kakkos et al., 2019) demonstrated significant alterations of alpha and theta band power, indicating increased workload (Hogervorst et al., 2014; Mühl et al., 2014; Brouwer et al., 2012) in a VR environment. Therefore, although these studies are valuable, they do not allow for the evaluation of the effectiveness of multisession training protocols. Another issue related to evaluating the effectiveness of cognitive training based on video games is their complexity due to the involvement of multiple cognitive and motor tasks, making it difficult to isolate the impact of the environment on a single task. To address the above issues, we designed an exploratory investigation aiming at a progress comparison of a gamified working memory task in a 2D computer screen and in head-mounted display virtual reality environments during three consecutive training sessions spread over 1 week. We monitored the course of neural, behavioral, and neuronal processing using EEG across the three sessions, including classical spectral and time-frequency analyses, spectral entropy, and neural connectivity estimated by Phase Locking Value. Additionally, we used theta to beta ratio to monitor changes of cognitive load over the sessions. Finally, we monitored behavioral performance using reaction times and accuracy of test performance.

## 2 Materials and methods

### 2.1 Experimental environments

**FIGURE 1:**
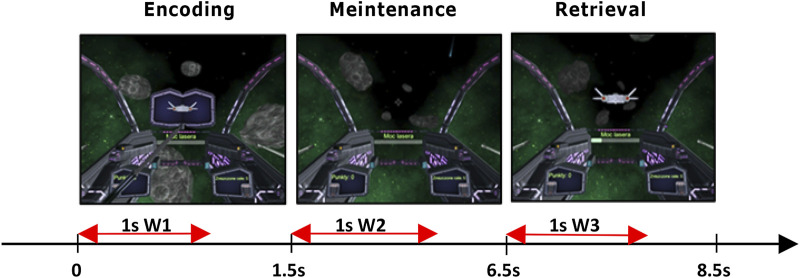
Delayed Match-To-Sample task: 1.5 s encoding phase, next 5 s to maintain the stimulus in working memory, and 2 s of a retrieval phase when participants pressed a button corresponding to the stimulus (left/right ctrl) that matched stimulus presented during encoding. In red are marked 1 s windows selected for EEG analysis.

Experiments for both groups were conducted in the same dimly lit room, at room temperature. For the 2D display group we used a standard 17 LCD monitor with participants sitting in comfortable chairs at a distance of approximately 50 cm from the screen. In the VR group, instead of monitors, participants were using Oculus Rift CV1 goggles. The field of view (FOV) for the computer screen was estimated as 40° and for goggles 90° , the VR version of the game included a detailed 270° view of the spaceship cabin and space beyond the spaceship, including stars and meteors. To ensure a similar difficulty level for both groups and mitigate potential artifacts caused by excessive head movements in the VR environment the shooting target appeared in the center of FOV (approximately 20° ) thus minimizing head movements in the VR environment and keeping a similar level of difficulty in the 2D one. The VR headset was worn over the EEG cap making sure that the harness of the headset did not press any electrode. To keep participants motivated and engaged throughout the test, the experimental task was adapted to an animated spacecraft shooting video game using the Unity programming environment for monitor screen and VR goggles. Otherwise both game variants were identical. Screenshots of the game are presented in Figure 1. Initial tests, performed before the main experiment, showed no effect of VR headset on EEG signal quality.

### 2.2 Procedure

For the cognitive procedure, we used the Delay Match-To-Sample (DMTS) task (Blough, 1959), which allows for studying working memory and attention in human subjects. We chose DMTS because it involves multiple memory functions, i.e., encoding, maintenance, and retrieval of stimulus representations in sequential order which allow for their individual analyses unlike the n-back task which requires only constant information updating (Ribeiro et al., 2019). In its basic version, the DMTS task comprised three phases: sample, delay, and choice. Each trial starts with the presentation of a sample to be memorized; next a delay was introduced. During the delay, participants were asked to maintain a representation of the sample. When the delay was over another stimulus or multiple stimuli were simultaneously presented to the participant. Participants were then asked to make a decision about does this stimulus match the sample presented in the first phase or does not. Our version of DMTS (Żygierewicz et al., 2022) started with a 2 s familiarization period, where no action from participants was required, followed by the sample encoding phase lasting 1.5 s, and then the animated silhouette of the spaceship was presented to participants. The delay phase–maintenance of the sample in memory, where no stimulus was presented lasted 5 s. During the last, 2 s long retrieval–choice phase, participants were presented with the spacecraft silhouette matching or not the sample. For analyses we chose 1 s window in each of the task phases. In the encoding and retrieval phases the window began at the time of stimulus appearance. In the maintenance phase window began 0.5 s after the end of the stimulus presentation to allow for fade-out of visual processing, which could interfere with memory retention processing readings. Window length was set to 1 s to cover the whole period of stimulus presentation. The duration of each phase was established experimentally during pilot trials to achieve the average performance of 70% accuracy. The screenshots and times for displaying individual trials are given in Figure 1. The entire experiment included 50 experimental trials involving matching and not matching the sample and were randomly intervened with 50 control trials that did not require attention. The times for displaying individual trials are given in Figure 1. To mitigate shooting to all targets, participants earned one point for each correct trial and lost one point for each incorrect one. Subjects from both groups performed the task in three repeated sessions (S1, S2, S3) on different days, 2–3 days apart.

### 2.3 Subjects

Twenty eight participants (13 women), aged 26–40 years who were not experienced game players were recruited through advertisements at local universities and employment agencies. The participants were randomized into VR and 2D display groups and performed three sessions of the adapted DMTS task during EEG recording. The EEG signal was recorded using Digitrack software (Elmiko l.t.d.) with 21 electrodes arranged in a 10–20 system, referenced to the right ear. All subjects provided informed written consent in accordance with the Helsinki Declaration as well as Institutional Review Board requirements.

### 2.4 Signal preprocessing

EEG signals were recorded at 500 Hz sampling rate. Artifact removal procedure was described in detail in (Żygierewicz et al., 2022). Preprocessing steps contained semi-automated methods, which included 0.5 Hz high-pass and 45 Hz low-pass filtering, baseline correction, exclusion of trials containing muscle artifacts, and independent component analysis (ICA). The identified eye movement and muscle artifacts components were removed. Next, for each channel and trial, we extracted three 1 s long windows (marked on Figure 1 as W1, W2, and W3) in order to investigate EEG signals properties in the encoding, maintenance, and retrieval phases of each task trial. If we set the beginning of the windows in each trial at time 0, the time spans of the three windows will encompass correspondingly: W1 [0, 1000 m], W2 [2000 m, 3000 m], W3 [6500 m, 7500 m]. In the last step, we extracted only epochs with correct responses, which were used in all subsequent analyses. Analysis was performed in the following classical EEG bands: delta ( δ ; 0.5–4 Hz); theta ( θ ; 4–7 Hz); alpha ( α ; 8–12 Hz); beta-1 ( β 1; 13–20 Hz); beta-2 ( β2 ; 21–30 Hz) and gamma ( γ ; 31–40 Hz). Spectral analyses included a comparison between groups of subjects of average power from the 1 s windows in the frequency bands. All power estimates were computed using MATLAB ‘pwelch’ function (with settings: 250 m segments with 50% overlap, windowed with a Hamming window). The obtained spectra were averaged in each window for a given channel and subject. For more detailed time-frequency analyses we used the ‘newtimef’ function implemented in EEGLAB (Delorme et al., 2011) in the described above windows and frequency bands. We applied Morlet wavelets decomposition using 3 cycles and 1 s long window with 200 time points.

Signal complexity: Complexity was evaluated by means of Spectral Entropy (SpEn) – theoretical information measure that provided an estimation of EEG regularity. SpEn definition was based on the formulation of Shannon’s entropy (Shannon, 1948), where the probability was replaced by the estimated power of spectral density PSD, Equation 1:

$$SpEn=-\frac{1}{log\left(L\right)}\overset{\mathrm{40Hz}}{\underset{f=1Hz}{\sum }}PSD\left(f\right)*log\left[PSD\left(f\right)\right]$$ (1)

where L was the number of spectral components.

Connectivity was assessed by Phase Locking Value (PLV) – a measure of phase dependence (Lachaux et al., 1999), here estimated between pairs of sites measured in separate frequency bands (defined above δ,θ,α,β1,β2 , and γ ) Equation 2;

$$PLV=\frac{1}{N}\overset{N}{\underset{n=1}{\sum }}exp\left(i\left(\phi_{1}\left[n\right]-\phi_{2}\left[n\right]\right)\right)$$ (2)

where $\phi_{1}$ and $\phi_{2}$ were phases of signal in a given frequency range for a pair of electrodes (1) and (2).

Attention and processing capacity: As the measure of cognitive processing capacity we used the θ/β ratio (TBR) Clarke et al. (2019). The θ/β ratio was calculated by dividing absolute theta power by absolute beta power ( β1 or β2 ) at each time window at the given trial and electrode site. To test differences between EEG signals in 2D monitor display and VR groups, the results of each measure were first averaged across all trials for each subject and channel in the three selected windows (W1, W2, W3). Next, a non-parametric unpaired Wilcoxon test with the significance threshold of p < 0.05 or p < 0.01 was used for each measure, and frequency band, and compared separately between the group of players in each game session. All data were analyzed in MATLAB (8.5.0, Math-Works, United States) using in-house scripts and EEGLAB toolbox (Delorme and Makeig, 2004).

## 3 Results

### 3.1 The game scores

**FIGURE 2:**
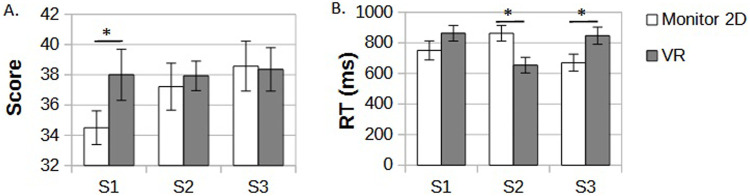
**(A)** An average number of correct answers in DMTS task in three consecutive sessions (S1-S3) in 2D monitor display and VR groups. **(B)** plot of average reaction time in the three sessions and separate 2D monitor display and VR groups. Significant differences are marked by black stars.

The VR group scored higher in the task compared to the 2D monitor display group, but only in the first game session Figure 2A. The average number of correct answers in the first session in the VR group was 38.1 vs. 34.5 in the 2D monitor group (*p* = 0.05). In the subsequent sessions, 2D monitor players gradually increased their scores to 37.2 in the second session and 38.5 in the third one, leveling up with results of VR players who maintained their results from the first session in the following ones at a relatively stable level (37.9 in session 2 and 38.2 in session 3). An average reaction time in session one showed a weak trend of faster correct answers for the VR group, but the results did not reach significance (p >= 0.1). For session 2, faster response was observed for 2D monitor display players and next in session 3 for the VR group (p < 0.05), as presented in Figure 2B.

### 3.2 EEG spectral analysis

**FIGURE 3:**
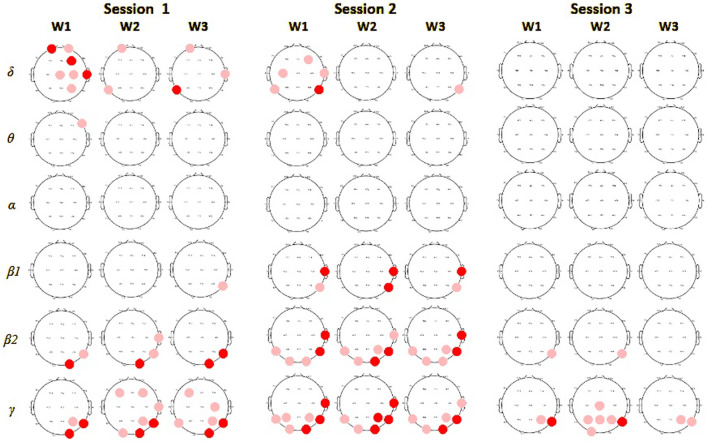
Spectral power comparison between players in monitor display and VR groups in session 1. In columns results for encoding (W1), maintenance (W2), and retrieval phases (W3), in rows for each of the frequency bands. Red circles mark electrodes with significantly higher values of average power in a particular frequency band for subjects in the VR group compared to the monitor display, deep red indicates a significance level at 0.01, and light red at 0.05.

Most prominent group differences in spectral analysis were found in the first session, in line with most behavioral differences. In the first session during the sample encoding phase, we observed significantly (p < 0.05) stronger spectral activity for the VR than the monitor display group, mostly in the δ band on frontal and right central EEG channels and in the β 2 and γ bands in the right posterior electrodes. During the maintenance and retrieval phases, the VR group revealed stronger activity in the δ band in one frontal and left parietal electrode and also more powerful activity in the β 2 and γ band, especially in the posterior and parietal areas on the right side. In the following sessions differences between groups gradually decreased, finally showing significant differences only in the posterior electrodes in β 2 and γ bands in the last session. The sites with significant spectral differences between groups of players are presented in Figure 3 in red.

**FIGURE 4:**
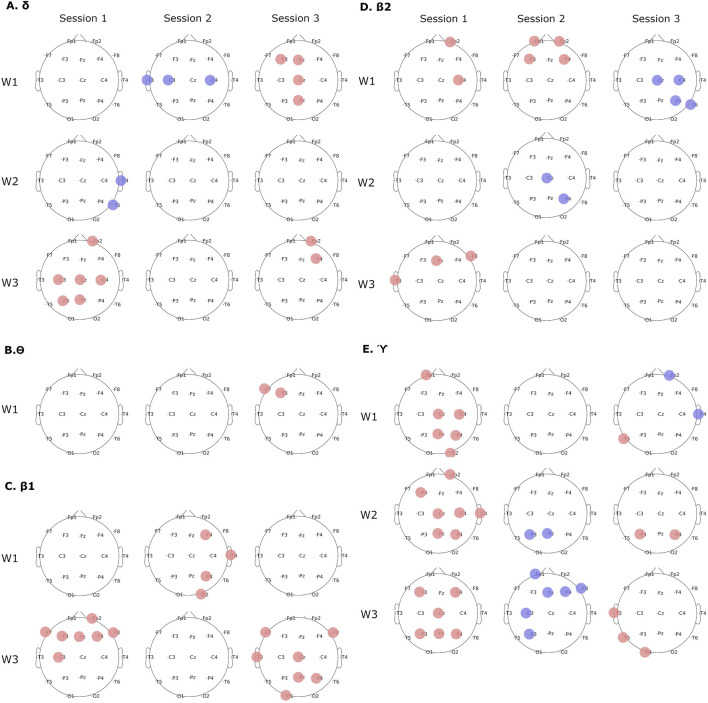
Group differences in time-frequency spectral analyses in subsequent sessions and task phases. **(A)** δ band, **(B)** θ band, **(C)** β 1 band, **(D)** β 2 band, **(E)** γ band. Red color denotes higher spectral power in the VR group, blue in the monitor group. Spectral bands or task phases where significant differences were not found are not shown or left empty. All differences significant at p < 0.01.

More detailed time-frequency analyses revealed group differences in all EEG bands except for α band. A most striking difference between analyzed groups was the higher spectral power of the VR group in session 1 in all EEG bands except for δ one where no differences were found (Figure 4). The number of electrodes showing group differences decreased in subsequent sessions. Most numerous differences showing higher spectral power of the VR group were found in the θ and γ bands in the encoding phase (Figures 4B, E). Higher spectral powers of the monitor display group were found only in the δ band in the maintenance phase in session 1 and the encoding phase in session 2 (Figure 4A and in the β 2 (Figure 4D) and γ bands (Figure 4E) in sessions 2 and 3.

**FIGURE 5:**
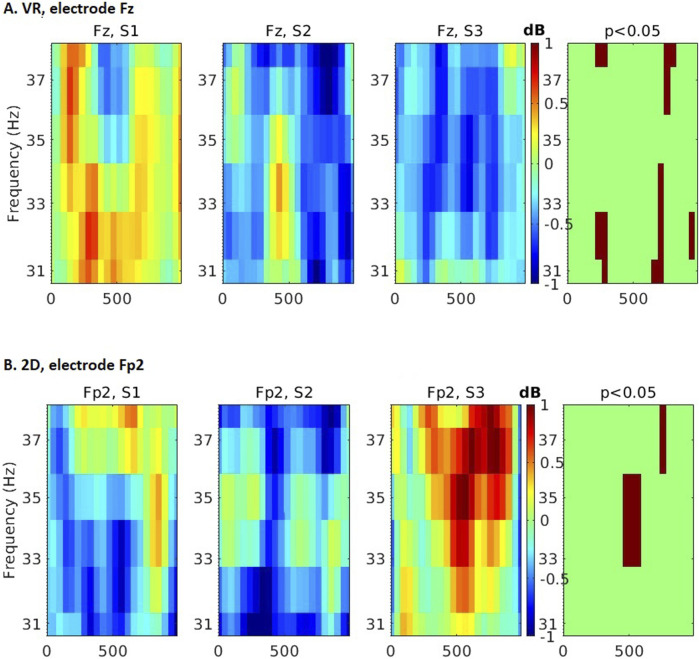
Time-frequency differences across 3 experimental sessions in the γ band of encoding phase. **(A)** VR group, an exemplary Fz electrode, **(B)** monitor display group, an exemplary Fp2 electrode. Main ANOVA effect (4th column) significant at p < 0.05.

Interestingly, comparison of EEG spectral changes which took place across sessions within each group revealed opposite patterns. In the γ band during the encoding phase in the VR group spectral power was highest in the first session and then decreased (p < 0.05, Figure 5A), while in the monitor display group lowest spectral power was observed in the first session and then increased (p < 0.05, Figure 5B).

### 3.3 Spectral entropy

**FIGURE 6:**
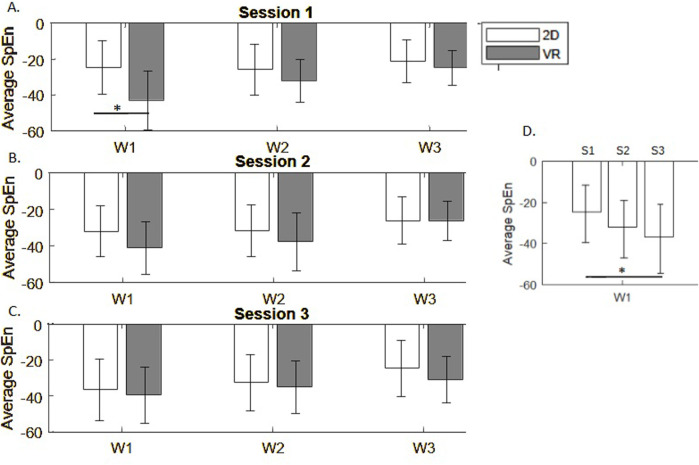
Spectral entropy differences across task phases in 3 experimental sessions **(A–C)**. **(D)** Average spectral entropy in the monitor display group in repeated sessions S1-S3.

First, we observed smaller EEG signal complexity in the VR group in the first session. Differences between monitor display and VR groups in average spectral entropy from all EEG channels were significant in encoding phase (*p* = 0.021), but not in maintenance (*p* = 0.16), and sample matching (*p* = 0.18) phases during the first session of the DMTS task (Figure 6A). This effect disappeared in sessions 2 (p > 0.2) and 3 (p > 0.33), (Figures 6B, C respectively) due to decrease of entropy in the monitor display group in repeated sessions as compared to session 1 (*p* = 0.002, Figure 6D).

### 3.4 Theta/beta ratio

**FIGURE 7:**
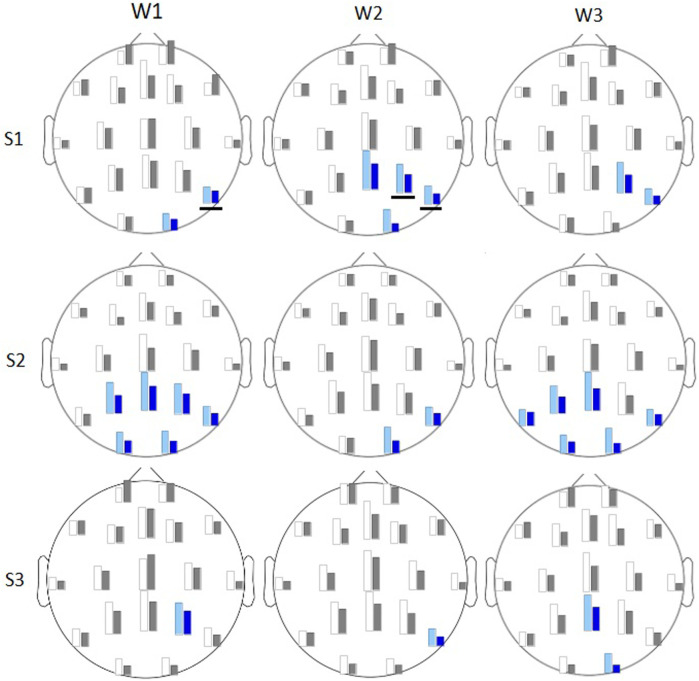
Average θ/β 2 ratios presented as bars for monitor display (white/light blue) and for VR (gray/dark blue) shown for 3 consecutive sessions S1-S3 (rows) and for windows W1-W3 (columns). For subjects in the 2D group electrodes with significantly higher values of average θ/β 2 ratio (on sig. level 0.05) are marked in blue, and those with the ratio higher on a significance level 0.01 are underlined.

In session 1 the θ/β 2 ratio was significantly smaller in VR than in the monitor group. The differences were found on posterior channels in all three phases of the task. Channels with significant differences in different task phases are shown in Figure 7. That was consistent with an indication of power spectra differences, where θ band did not show significant differences between the groups of analysis, and β 2 showed higher power for the VR group (Figure 3). In session 2 VR players showed lower θ/β 2 ratio in posterior channels in comparison to monitor display group (Figure 7B), mostly in encoding and retrieval phases. In session 3 we observed further reduction of this effect (Figure 7).

### 3.5 EEG connectivity

**FIGURE 8:**
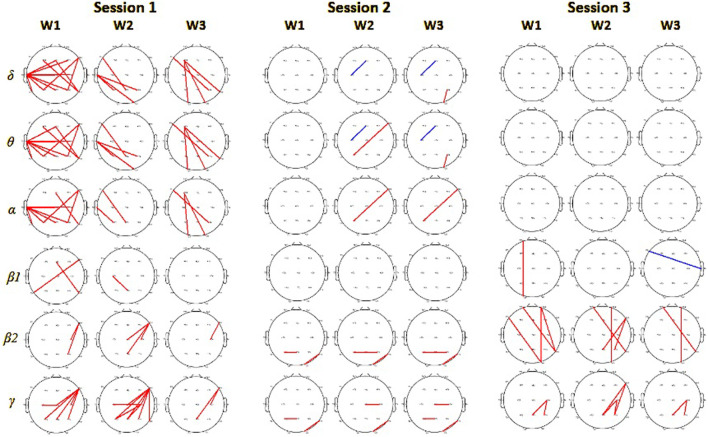
Phase locking value connectivity differences between monitor and VR groups in 3 consecutive sessions. From left for session 1, session 2, and session 3. Red colors mark pairs of electrodes with significantly higher values for subjects in the VR group (on significance level 0.01), and blue for the monitor display group.

EEG signals interrelations, measured by PLV, revealed in session 1 stronger connectivity in the VR group of subjects compared to the monitor group. That was observed especially in δ , θ , and α frequencies in left centro-parietal sites during all three analyzed phases of the DMTS task. For higher frequencies, we noted significantly stronger connections between left frontal and centro-parietal regions for VR subjects (Figure 8). This effect, similar to spectral differences, was not observed in sessions 2 and 3 for which the only differences between VR and monitor display groups were observed for a few connections in higher frequencies stronger for VR group.

## 4 Discussion

Results of our exploratory study comparing 2D monitor display and VR groups in three game sessions showed group differences only in the first session. The VR group members outperformed the monitor display group in the first session and maintained their performance level throughout the remaining two, while the monitor display group increased performance in each session, eventually leveling up with the VR group. The studies comparing behavioral and neurophysiological effects of the VR and computer screen-based tests over multiple training sessions are very rare. One of the very few such investigations is the study performed by Berger and colleagues (Berger et al., 2022), who investigated the effect of neurofeedback training in VR and 2D environments on SMR power. Although their results pointed to a linear increase of the SMR power only in the VR groups, the graphical implementation of the task in both environments was different, therefore one can not exclude that the effect was due to differences in visual stimulus. The study also did not investigate putative differences in cognitive performance. From that perspective more interesting seems to be the experiment conducted by Barrett et al. (2022). In this experiment three groups of participants performed identical categorization tasks also in terms of the visual stimulus implemented in the three environments: VR its computer screen version and the flat version also on a computer screen. The first and second task implementations exactly match the settings of our experiment, however the cognitive task was different. Although this study included only one session, the performance differences in our study were found only in the first session. Interestingly, Barrett et al. (2022) found no behavioral differences between the groups, but the number of fixations in the VR environment was significantly higher than in the 2D one. Also other, previous experiments comparing monitor display and VR environments in a single session showed no benefit of greater immersion experience elicited by VR environment (Pallavicini and Pepe, 2019; Buttussi and Chittaro, 2017; Lugrin et al., 2013). Our seemingly contradictory behavioral performance results may stem from specific game design solely based on a simplified Delay Match-to-Sample task. Games utilizing more selective cognitive functions could be more sensitive to the environmental effects than more complex games, which require more variability and demand on cognitive functions than one function. Another explanation of these contradictions could be possible differences in the interactions between the task at hand and the environment.

To further investigate the effect of the game environment on memory performance we analyzed EEG data. The pattern of behavioral group differences was followed by EEG activity. Analyses of the spectral power differences across sessions revealed two prominent observations: first, higher power of the δ band in the VR group (in the relation to monitor display group) in the encoding phase of the first session, which gradually diminished in subsequent sessions; and second, differences in the higher frequency bands mostly in the second session in posterior locations. Although δ band activity is primarily associated with deep sleep, some studies also reported relationships between δ band activity and cognitive task performance. The work of Mathewson et al. (2012), found a positive correlation between δ band activity in the frontal and parietal electrodes and learning rate in video games. Increased δ band activity was also found to be correlated with higher attention to internal processes (Harmony et al., 1996). These findings may suggest that observed game performance differences could arise from faster memory learning rate elicited by increased attention to internal processes in the VR group. In subsequent sessions, participants from the monitor group would gradually adapt to the game environment, improving their learning rate through increased attention to internal processes leveraging their learning rate as indicated by diminished differences in δ band activity. Indeed, more detailed time-frequency analyses revealed increased δ power in the second session in the monitor display group in frontal electrodes, while there were no differences in the VR group. The notion of the VR effect on the attention state in the first session can be further confirmed by the results of spectral entropy analyses. Spectral entropy is often considered in the context of attention (Ke et al., 2014; Lesenfants and Francart, 2020; Lesenfants et al., 2018) pointing out that the state of the attention can be reliably detected using this method. Our results showed lower spectral entropy in the VR group than in the monitor display group in the encoding phase in session 1. In the remaining two sessions spectral entropy of the monitor display group gradually decreased, reaching a significant difference against the first session in session 3 and leveling with the VR group, in which entropy level was stable over all three sessions. Interestingly, all spectral differences between the VR and monitor display groups were found in the encoding phase, suggesting a positive effect of the VR environment in this particular phase of working memory processing. This observation can be further confirmed by stronger, left hemisphere theta band connectivity most pronounced in the encoding phase as revealed by PLV analyses. Summerfield and Mangels (2005) found that item-context binding during the encoding phase is mediated by fronto-posterior EEG phase locking within and between hemispheres in the θ band, also Weiss et al. (2000) observed increased θ band synchronizations during successful encoding of concrete and abstract nouns. Strong fronto-parietal reciprocal coupling during Working Memory tasks (visual and auditory), in different frequencies, especially θ , γ was also confirmed in the work of Blinowska et al. (2013). Finally, Sato and Yamaguchi (2007) found that θ coherence increased during successful encoding of the object–place associations. Our second observation concerned the activity in higher EEG frequencies ( β and γ ). Spectral analyses of the VR and monitor display groups revealed higher activity in the VR participants mostly in the maintenance phase in session 1 and in all task phases in session 2. Several studies indicated the role of higher frequency EEG bands in the maintenance phase of working memory. Notably, Fernández et al. (2021) found an increase of β activity in occipital and occipito-temporal regions during the delay period of DMS tasks. Also, Pavlov and Kotchoubey (2022) posited that β activity is related to the maintenance of object representations in working memory. More detailed time-frequency analyses showed higher spectral power in the γ band in the monitor display group in session 2 in the maintenance and retrieval phases, which may indicate that participants of this group adapted to the game environment. The better performance of the VR group in session 1 may also be explained by the higher spectral power in that group in the θ and γ bands in the encoding phase, as revealed by time-frequency analysis. θ and γ bands were found to constitute a working memory mechanism (Alekseichuk et al., 2016; Chaieb et al., 2015; Roux and Uhlhaas, 2014; Kamiński et al., 2011) and higher spectral power of those bands in the encoding phase of VR group may indicate higher performance of their short term memory processes resulting from attention to internal processes (Harmony et al., 1996). Finally, our study revealed that subjects in the monitor display group exhibited significantly higher average θ/β 2 (21–30 Hz) ratios than those in the VR group. The θ/β ratio was initially thought to reflect the arousal mechanism, but subsequent research has suggested that it represents cognitive processing capacity (Clarke et al., 2019) and serves as an objective indicator of executive cognitive control, particularly attention control, in healthy adults. The θ/β ratio, which is the ratio of fronto-central θ (4–7 Hz) to β oscillations (13–30 Hz), has been found to be negatively correlated with attentional control, reinforcement learning, executive function, and age (Finley et al., 2022). Therefore, the higher θ/β 2 (21–30 Hz) ratio in the monitor display group suggests that the group requires higher mobilization of attention, especially during the maintenance phase. However, observed in our study differences in EEG activity may indicate faster learning rate in the VR environment than in the 2D one; the differences did not survive multiple comparison correction which indicate rather low effect. An important limitation of our study pertains to the insufficient control over the benefits derived from the stereoscopy effect. Introducing an additional experimental group could elucidate whether the effects noted in the initial session were a result of the experienced immersion in the VR due to stereoscopy or a large visual display spanning most of the field of view. Future research should also consider expanding the number of sessions to approximately 10–15 to better understand the long-term impacts of VR training. However, given the trend of diminishing differences between the tested groups observed in our study, we propose that any long-term effects of VR training could, at most, be moderate.

## 5 Conclusion

Our findings show that the impact of the VR environment may be differentiated for various cognitive functions and the benefits are primarily limited to the learning rate. Therefore, further studies comparing behavioral results and learning rates for different cognitive functions are needed before any clinical applications. Another conclusion that could be drawn from this study is that observed differences in brain activity induced by different task environments do not necessarily result in desired behavioral improvements.

## Data Availability

The raw data supporting the conclusions of this article will be made available by the authors, without undue reservation.
