# Corrigendum: Performance of game sessions in VR vs standard 2D monitor environment. An EEG study

**DOI:** 10.3389/fphys.2025.1616912

**Published:** 2025-05-09

**Authors:** Urszula Malinowska, Jakub Wojciechowski, Marek Waligóra, Jacek Rogala

**Affiliations:** ^1^ Institute of Experimental Physics, University of Warsaw, Warsaw, Poland; ^2^ Nencki Institute of Experimental Biology, Warsaw, Poland; ^3^ Bioimaging Research Center, Institute of Physiology and Pathology of Hearing, Kajetany, Poland; ^4^ Faculty of Physics, University of Warsaw, Warsaw, Poland

**Keywords:** virtual reality, EEG, DMTS, cognitive rehabilitation, time-frequency

In the published article, there was an error in the **Funding** statement. The **Funding** statement was displayed as “The author(s) declare that financial support was received for the research, authorship, and/or publication of this article. This work was supported by the National Centre for Research and Development grant POIR 01.01.01-00-178/15”. The correct **Funding** statement appears below.

**Funding**

The author(s) declare that no financial support was received for the research and/or publication of this article.

In the published article, there was an error regarding the affiliation for Jacek Rogala. Instead of having affiliation 1, they should have Faculty of Physics, University of Warsaw, Warsaw, Poland.

The authors apologize for these errors and state that these do not not change the scientific conclusions of the article in any way. The original article has been updated.

